# TRPM7 channel inhibition exacerbates pulmonary arterial hypertension through MEK/ERK pathway

**DOI:** 10.18632/aging.102036

**Published:** 2019-06-19

**Authors:** Junhui Xing, Mengyu Wang, Jin Hong, Yueqiao Gao, Yuzhou Liu, Heping Gu, Jianzeng Dong, Ling Li

**Affiliations:** 1 Department of Cardiology, The First Affiliated Hospital of Zhengzhou University, Zhengzhou, China; 2 Department of Endocrinology, The First Affiliated Hospital of Zhengzhou University, Zhengzhou, China

**Keywords:** pulmonary arterial hypertension, TRPM7, pulmonary artery smooth muscle cell, proliferation, apoptosis resistance

## Abstract

Cellular senescence is an important mechanism of autonomous tumor suppression, while its consequence such as the senescence-associated secretory phenotype (SASP) may drive tumorigenesis and age-related diseases. Therefore, controlling the cell fate optimally when encountering senescence stress is helpful for anti-cancer or anti-aging treatments. To identify genes essential for senescence establishment or maintenance, we carried out a CRISPR-based screen with a deliberately designed single-guide RNA (sgRNA) library. The library comprised of about 12,000 kinds of sgRNAs targeting 1378 senescence-associated genes selected by integrating the information of literature mining, protein-protein interaction network, and differential gene expression. We successfully detected a dozen gene deficiencies potentially causing senescence bypass, and their phenotypes were further validated with a high true positive rate. RNA-seq analysis showed distinct transcriptome patterns of these bypass cells. Interestingly, in the bypass cells, the expression of SASP genes was maintained or elevated with *CHEK2*, *HAS1*, or *MDK* deficiency; but neutralized with *MTOR*, *CRISPLD2*, or *MORF4L1* deficiency. Pathways of some age-related neurodegenerative disorders were also downregulated with *MTOR*, *CRISPLD2*, or *MORF4L1* deficiency. The results demonstrated that disturbing these genes could lead to distinct cell fates as a consequence of senescence bypass, suggesting that they may play essential roles in cellular senescence**.**

## INTRODUCTION

Pulmonary arterial hypertension (PAH) is pathologically featured by the narrowing and obliteration of small pulmonary arteries [1]. Consequently, the increase in pulmonary vascular resistance causes a progressive elevation of pulmonary artery pressure that ultimately leads to death from right heart failure [2, 3]. PAH affects approximate 100 million people in the world, and the mortality remains high due to ineffective therapies and inevitable disease progression in most patients [4, 5]. Thus, there is an urgent need to identify new therapeutic targets for PAH.

During the pathogenesis of PAH, the increased proliferation and apoptosis resistance of endothelial cells (ECs), smooth muscle cells (SMCs), and fibroblasts accelerate the remodeling of pulmonary arteries [6]. The physiological abnormalities, including the upregulation of growth factors and metabolic changes, render the vascular wall of pulmonary arteries a pro-proliferative and anti-apoptotic microenvironment in PAH patients [7–9]. Therefore, it might be unsurprising that anti-neoplastic drugs capable of inhibiting proliferation and reversing apoptosis resistance show beneficial effects in PAH treatment [10, 11]. We hypothesized that proteins involved in the regulation of proliferation and apoptosis of pulmonary artery cells may influence PAH pathogenesis and that their targeting might provide a new option for PAH therapy.

The transient receptor potential melastatin 7 (TRPM7) belongs to TRP channel superfamily [12]. TRPM7 acts as a magnesium channel and regulates cellular magnesium signaling and homeostasis in mammalian cells [13]. A growing number of studies have associated TRPM7 with the pathophysiology of several diseases, such as cancer, ischemic stroke cardiac fibrogenesis, Parkinson's disease, etc, [14–17]. In the recent decade, TRPM7-mediated magnesium influx has been shown to affect cellular activities implicated in hypertension, such as endothelial dysfunction and vascular integrity maintenance [18]. In vascular smooth muscle cells (VSMCs) of spontaneous hypertensive rats, TRPM7 expression and intracellular magnesium are decreased [19]. But the specific contribution of TRPM7 to hypertension is still ambiguous. Moreover, TRPM7 was found to promote the proliferation of VSMCs and vascular endothelial cells [20, 21]. These clues motivated us to investigate whether TRPM7 is involved in PAH pathogenesis.

To date, multiple in vivo preclinical PAH models, albeit with differential limitations, have been developed to mimic disease pathogenesis in human and used to decipher molecular mechanisms and test the efficacy of potential drugs, and among them, the model induced in Sprague-Dawley rats via chronic exposure to hypoxia has been commonly utilized [22]. In this study, we demonstrate that the inhibition of TRPM7 promotes proliferation and apoptosis resistance in PASMCs in vitro and exacerbates PAH in hypoxia-induced rat model, in which the activation of MEK/ERK pathway represents a prominent mechanism, therefore providing a new insight into PAH pathogenesis and implicating a potential for developing novel therapies.

## RESULTS

### TRPM7 downregulation in PAH PASMCs from human and an animal model

**Figure 1:**
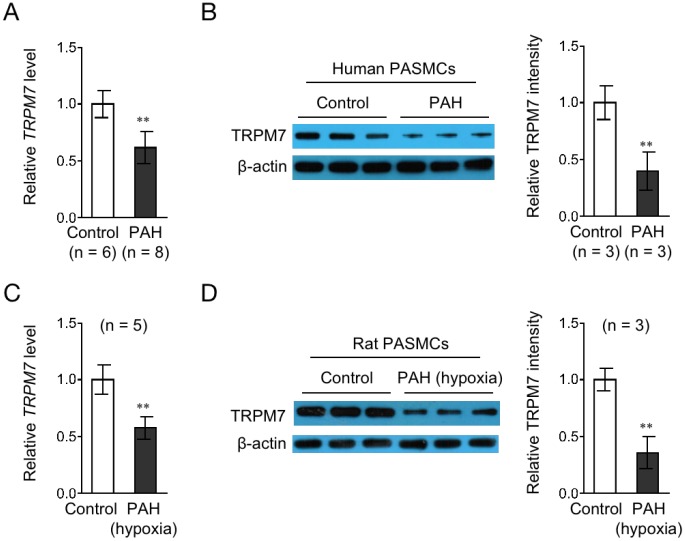
**TRPM7 expression is downregulated in PASMCs from PAH human and a rat model.** (**A**) The mRNA level of TRPM7 in PASMCs from control (n = 6) or PAH (n = 8) human donors was determined by qRT-PCR analysis. β-actin was used as a reference control. Data are mean ± SD. Unpaired Student’s *t*-test. **, P < 0.01 compared to control. (**B**) The protein level of TRPM7 in PASMCs from 3 representative control and PAH human donors was determined by Western blot analysis. β-actin was used as a loading control. The representative images (left) and band intensity analysis (right) are shown. Data are mean ± SD. Unpaired Student’s *t*-test. **, P < 0.01 compared to control. (**C**) The mRNA level of TRPM7 in PASMCs from control (n = 5) or hypoxia-induced PAH (n = 5) rats was determined by qRT-PCR analysis. β-actin was used as a reference control. Data are mean ± SD. Unpaired Student’s *t*-test. **, P < 0.01 compared to control. (**D**) The protein level of TRPM7 in PASMCs from 3 representative control and hypoxia-induced PAH rats was determined by Western blot analysis. β-actin was used as a loading control. The representative images (left) and band intensity analysis (right) are shown. Data are mean ± SD. Unpaired Student’s *t*-test. **, P < 0.01 compared to control.

Although TRPM7 has been implicated in vascular pathological changes that underlie hypertension [18, 23], whether it is associated with PAH pathogenesis remains unknown. To investigate this issue, we initially examined its expression in PASMCs obtained from PAH patients and control donors. qRT-PCR analysis showed that compared with PASMCs from control donors, the mRNA level of TRPM7 was significantly downregulated in PASMCs from PAH patients (Figure 1A). Similarly, the protein expression of TRPM7 was also deceased in PAH PASMCs, as analyzed by Western blotting (Figure 1B). To broaden this finding, we next checked whether similar trend of TRPM7 expression in PASMCs could be reproduced in a rat PAH model. Indeed, as shown in Figure 1C–1D, compared with those in PASMCs from control lung, both the mRNA level and protein level of TRPM7 were decreased in PASMCs from rats with hypoxia-induced PAH. Thus, these results show that TRPM7 expression is downregulated in PASMCs from both human and a rat model with PAH, and also implicate that TRPM7 downregulation may have relevance to PAH pathogenesis.

### TRPM7 downregulation in PASMCs in response to PAH stimuli in vitro

**Figure 2:**
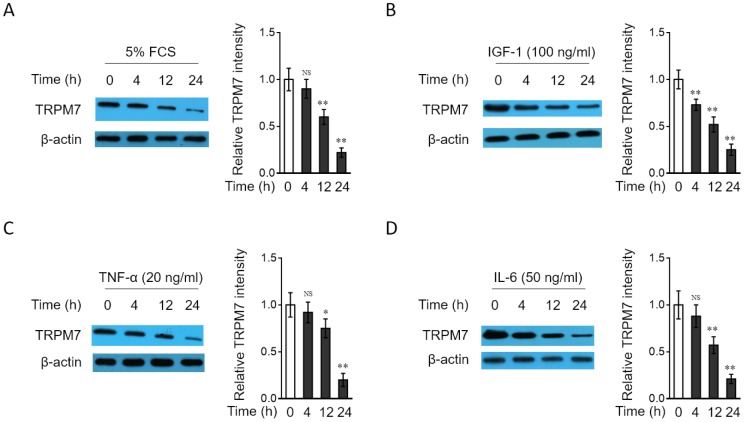
**TRPM7 expression is downregulated in PASMCs treated with PAH stimuli in vitro** (**A**–**D**) PASMCs from rats were serum starved for 24 h, followed by incubation with medium alone (control) or containing 5% fetal calf serum (FCS) (**A**), 100 ng/ml IGF-1 (**B**), 20 ng/ml TNF-α (**C**), or 50 ng/ml IL-6 (**D**) for increasing time periods as indicated. The protein level of TRPM7 was determined by Western blot analysis. β-actin was used as a loading control. The representative images (left) and band intensity analysis (right) are shown. Results are representative of 3 independent experiments. Data are mean ± SD. One-way ANOVA test. **, P < 0.01; *, P < 0.05; NS, not significant, as compared to control.

To test whether the downregulation of TRPM7 expression in PAH PASMCs is related to the stimulation by PAH stimuli, we cultured the rat normal PASMCs in vitro in medium containing various relevant PAH stimuli, such as 5% fetal calf serum (FCS) or different growth factors and cytokines, including IGF-1, TNF-α or IL-6, all of which have been shown to be pro-hypertensive [44]. As results, we found that the expression of TRPM7 in PASMCs was decreased in a time-dependent manner when treated with 5% FCS (Figure 2A), IGF-1 (Figure 2B), TNF-α (Figure 2C) and IL-6 (Figure 2D). These data not only suggest that TRPM7 expression in PASMCs could be downregulated in response to PAH stimuli, at least in vitro condition, but also hint that TRPM7 downregulation in PAH PASMCs may be caused by the stimulation of PAH stimuli present in focal sites.

### PAH stimuli reduce TRPM7 currents and free magnesium concentration in PASMCs

**Figure 3:**
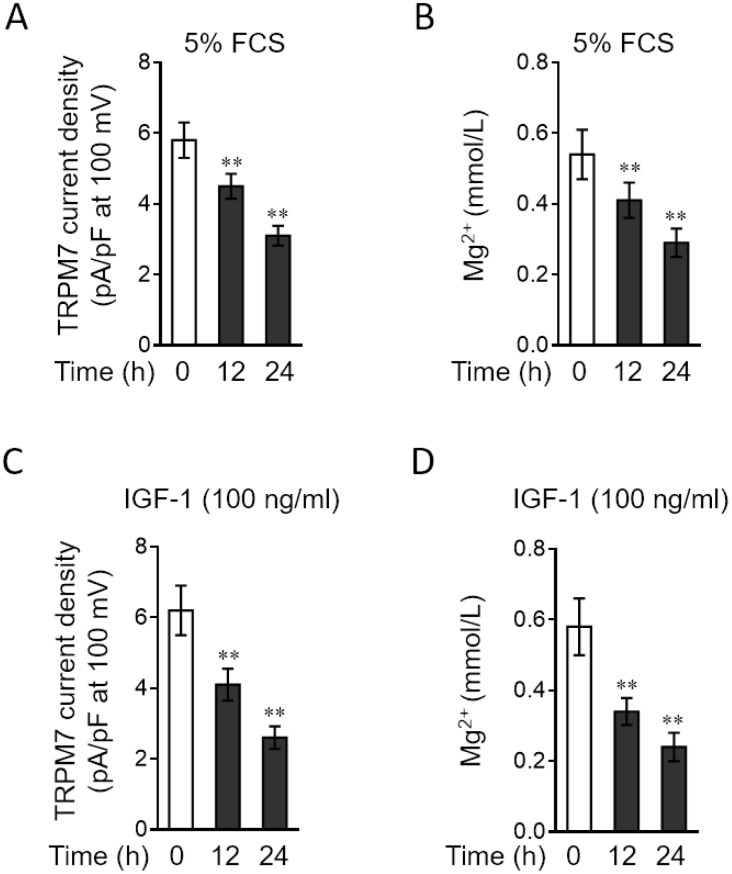
**PAH stimuli reduce TRPM7 currents and intracellular free Mg2 concentration in PASMCs in vitro.** (**A**–**B**) PASMCs from rats were serum starved for 24 h, followed by incubation with medium alone (control) or containing 5% fetal calf serum (FCS) for 12 or 24 h. (**A**) TRPM7 currents were recorded using the whole-cell patch-clamp technology with ramp from -100 mV to 100 mV. The TRPM7 currents density (pA/pF) with ramp at 100 mV is shown. Fifty cells were analyzed in each treatment. Data are mean ± SD. One-way ANOVA test. **, P < 0.01 compared to control. (**B**) Intracellular free Mg^2+^ concentration was determined. Data are mean ± SD from 3 independent experiments. One-way ANOVA test. **, P < 0.01 compared to control. (**C**–**D**) PASMCs from rats were serum starved for 24 h, followed by incubation with medium alone (control) or containing 100 ng/ml IGF-1 for 12 or 24 h. The TRPM7 currents density (**C**) and intracellular free Mg^2+^ concentration (**D**) were determined and analyzed as in (**A**–**B**).

TRPM7 functions as a cation channel that promotes vascular magnesium transport and homeostasis [24, 25]. Since TRPM7 expression is downregulated in PASMCs stimulated by relevant PAH stimuli, we asked whether TRPM7-mediated magnesium transport is also impaired in PASMCs under these conditions. To test this possibility, we applied the whole-cell patch-clamp technique to record TRPM7 currents and also compared free intracellular magnesium concentration in PASMCs treated with or without PAH stimuli [26]. The result showed that compared with normal medium culture, 5% FCS stimulation caused a significant reduction in the density of TRPM7 currents (Figure 3A). Consistent with this, the free intracellular magnesium concentration in PASMCs was also decreased when stimulated with 5% FCS (Figure 3B). Moreover, similar results were obtained when PASMCs were stimulated with IGF-1, another PAH stimulus (Figure 3C–3D). Together, these observations indicate that along with TRPM7 downregulation, magnesium transport is also attenuated in PASMCs in response to PAH stimuli, suggesting a functional impairment of TRPM7 associated with PAH.

### TRPM7 inhibition or knockdown increases proliferation and apoptosis resistance in PASMCs

**Figure 4:**
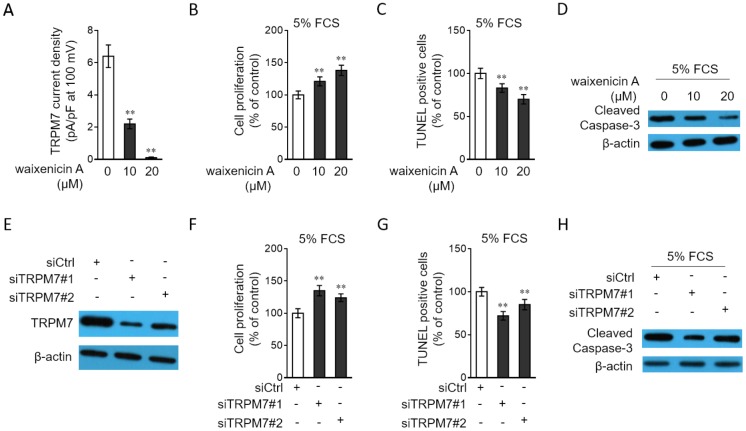
**TRPM7 inhibition or knockdown promotes proliferation and apoptosis resistance in PASMCs.** (**A**) PASMCs from rats were treated with vehicle control, 10 μM or 20 μM waixenicin A for 24 h. TRPM7 currentss were recorded using the whole-cell patch-clamp technology with ramp from -100 mV to 100 mV. TRPM7 currents density (pA/pF) with ramp at 100 mV is shown. Fifty cells were analyzed in each treatment. Data are mean ± SD. One-way ANOVA test. **, P < 0.01 compared to control. (**B**–**D**) PASMCs from rats were serum starved for 24 h, followed by incubation with medium containing 5% FCS for 24 h in the presence or absence of 10 μM or 20 μM waixenicin A. (**B**) Cell proliferation was determined by BrdU incorporation assay. (**C**) Cell apoptosis was detected by TUNEL staining. Results are expressed as a percentage relative to control. Data are mean ± SD. n = 3. One-way ANOVA test. **, P < 0.01 compared to control. (**D**) The protein expression of cleaved caspase-3 was determined by Western blot analysis. β-actin was used as a loading control. The representative images from 3 independent experiments are shown. (**E**) PASMCs from rats were transfected with siRNA control, siRNA TRPM7#1 or siRNA TRPM7#2. After 72 h of transfection, the protein level of TRPM7 was determined by Western blot analysis. β-actin was used as a loading control. The representative images from 3 independent experiments are shown. (**F**–**H**) PASMCs from rats were transfected with siRNA control, siRNA TRPM7#1 or siRNA TRPM7#2. After 24 h of transfection, cells were serum starved for 24 h, followed by incubation with medium containing 5% FCS for another 24 h. Cell proliferation (**F**), cell apoptosis (**G**) and the protein expression of cleaved caspase-3 (**H**) were determined and analyzed as in (**B**–**D**).

TRPM7 downregulation in PAH PASMCs led us to explore whether it affects the proliferation and apoptosis of PASMCs, two hallmarks of PASMCs in PAH [27, 28]. To address this issue, waixenicin A, a potent and relatively specific inhibitor of TRPM7 ion channels [29, 30], was used to block its function. The inhibition of TRPM7 function by waixenicin A was confirmed by the drastically decreased density of TRPM7 currents, as compared with vehicle control (Figure 4A). The stimulative effects of 5% FCS on the proliferation (Supplementary Figure 1A) and apoptosis resistance (Supplementary Figure 1B) in PASMCs were firstly validated. Next, the result from BrdU incorporation assay showed that waixenicin A treatment significantly increased the proliferation of PASMCs stimulated with 5% FCS (Figure 4B). Further, PASMCs were stimulated with 5% FCS in the presence or absence of waixenicin A, and then apoptosis was detected by TUNEL assay. We found that waixenicin A treatment significantly reduced the number of apoptotic cells (Figure 4C). Consistently, as shown by Western blotting analysis, waixenicin A treatment also decreased the expression level of cleaved caspase-3 in PASMCs (Figure 4D). Furthermore, notably, Mg^2+^ anaplerosis via exogenous supplementation largely rescued the effects of waixenicin A treatment on the proliferation and apoptosis resistance in PASMCs (Supplementary Figure 2A–2C). Hence, these results describe that TRPM7 inhibition with waixenicin A increases proliferation and apoptosis resistance in PASMCs under the stimulation of 5% FCS in vitro, which could be at least in part attributed to impairment of Mg^2+^ intake.

To confirm the above observations, we depleted TRPM7 expression through siRNA transfection. Two independent siRNAs with different sequences targeting TRPM7 were used (siTRPM7#1 and siTRPM7#2). Contrary to the scrambled control, these two independent siRNAs displayed high efficiency in silencing TRPM7 expression in PASMCs (Figure 4E). Moreover, in concert with results obtained from waixenicin A treatment (Figure 4B–4C), TRPM7 knockdown increased the proliferation (Figure 4F) and reduced apoptosis (Figure 4G–4H) of PASMCs. Collectively, these lines of evidence show that pharmaceutical inhibition or knockdown of TRPM7 increases proliferation and apoptosis resistance in PASMCs, which may provide a functional relevance of TRPM7 downregulation to PAH pathogenesis.

### TRPM7 overexpression decreases proliferation and apoptosis resistance in PASMCs

**Figure 5:**
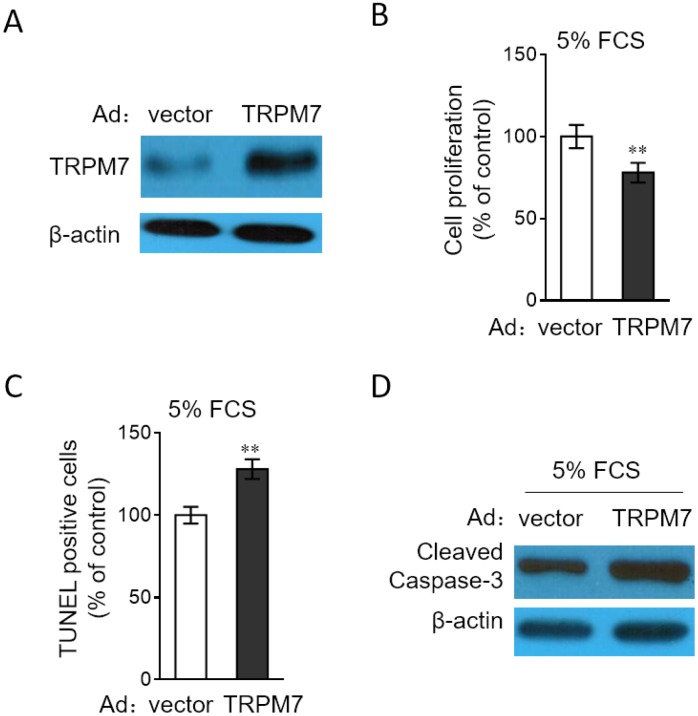
**TRPM7 overexpression reverses proliferation and apoptosis resistance in PASMCs**. (**A**) PASMCs from rats were infected with adenovirus expressing control vector (Ad-Ctrl) or TRPM7 (Ad-TRPM7). After 72 h of infection, the protein level of TRPM7 was determined by Western blot analysis. β-actin was used as a loading control. The representative images from 3 independent experiments are shown. (**B**–**D**) PASMCs from rats were infected with Ad-Ctrl or Ad-TRPM7. After 24 h of infection, cells were serum starved for 24 h, followed by incubation with medium containing 5% FCS for another 24 h. (**B**) Cell proliferation was determined by BrdU incorporation assay. (**C**) Cell apoptosis was detected by TUNEL staining. Results are expressed as a percentage relative to control. Data are mean ± SD. n = 3. One-way ANOVA test. **, P < 0.01 compared to vector. (**D**) The protein expression of cleaved caspase-3 was determined by Western blot analysis. β-actin was used as a loading control. The representative images from 3 independent experiments are shown.

In order to consolidate the function of TRPM7 in PASMCs, we performed the enforced overexpression of TRPM7 in PASMCs through adenovirus infection. Adenovirus-mediated overexpression of TRPM7 in PASMCs was first verified by Western blotting analysis (Figure 5A). Consequently, we discovered that in contrast to TRPM7 inhibition or knockdown, TRPM7 overexpression decreased cell proliferation (Figure 5B) and meanwhile, increased the number of apoptotic cells (Figure 5C) and expression of cleaved caspase-3 (Figure 5D) in PASMCs stimulated with 5% FCS, therefore reinforcing the notion that TRPM7 functions as a negative regulator of proliferation and apoptosis resistance in PASMCs.

### TRPM7 inhibition or knockdown increases proliferation and apoptosis resistance in PASMCs through MEK/ERK pathway

**Figure 6:**
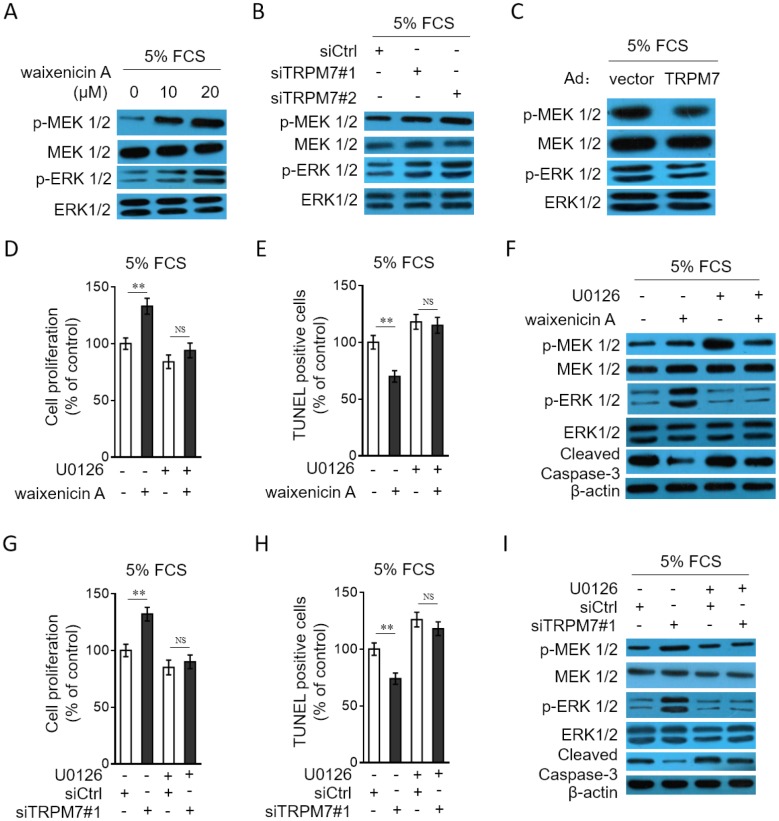
**TRPM7 inhibition or knockdown promotes PASMCs proliferation and apoptosis resistance through MEK/ERK pathway.** (**A**) PASMCs from rats were serum starved for 24 h, followed by incubation with medium containing 5% FCS for 24 h in the presence or absence of 10 μM or 20 μM waixenicin A. The protein expression of p-MEK 1/2, MEK 1/2, p-ERK 1/2 and ERK 1/2 was determined by Western blot analysis. (**B**) PASMCs from rats were transfected with siRNA control, siRNA TRPM7#1 or siRNA TRPM7#2. After 24 h of transfection, cells were serum starved for 24 h, followed by incubation with medium containing 5% FCS for another 24 h. The protein expression of p-MEK 1/2, MEK 1/2, p-ERK 1/2 and ERK 1/2 was determined by Western blot analysis. (**C**) PASMCs from rats were infected with Ad-Ctrl or Ad-TRPM7. After 24 h of infection, cells were serum starved for 24 h, followed by incubation with medium containing 5% FCS for another 24 h. The protein expression of p-MEK 1/2, MEK 1/2, p-ERK 1/2 and ERK 1/2 was determined by Western blot analysis. β-actin was used as a loading control. The representative images from 3 independent experiments are shown. (**D**–**F**) PASMCs from rats were serum starved for 24 h, followed by incubation with medium containing 5% FCS for 24 h in the presence or absence of 20 μM waixenicin A or 10 μM U0126. (**D**) Cell proliferation was determined by BrdU incorporation assay. (**E**) Cell apoptosis was detected by TUNEL staining. Results are expressed as a percentage relative to control. Data are mean ± SD. n = 3. Unpaired Student’s *t*-test. **, P < 0.01; NS, not significant, as compared to control. (**F**) The protein expression of targets as indicated was determined by Western blot analysis. β-actin was used as a loading control. The representative images from 3 independent experiments are shown. (**G**–**I**) PASMCs from rats were transfected with siRNA control, siRNA TRPM7#1. After 24 h of transfection, cells were serum starved for 24 h, followed by incubation with medium containing 5% FCS for another 24 h in the presence or absence of 10 μM U0126. Cell proliferation (**G**), cell apoptosis (**H**) and the protein expression of targets (**I**) was determined and analyzed as in (**D**–**F**).

To gain a mechanistic insight into the molecular mechanisms by which TRPM7 affects the proliferation and apoptosis resistance in PASMCs, we focused on MEK/ERK pathway, since this pathway plays a critical role in regulating cell proliferation and apoptosis [31], and it’s also reported that this pathway mediates TRPM7 effect on vascular cell proliferation [20, 21]. Compared with normal culture, in PASMCs cultured with 5% FCS, the MEK/ERK pathway was activated (Supplementary Figure 3). We then tested whether TRPM7 manipulation affects the activation of MEK/ERK pathway in PASMCs. As shown, TRPM7 inhibition with waixenicin A (Figure 6A) or expression knockdown by siRNA transfection (Figure 6B) promoted the phosphorylation of MEK and ERK in PASMCs stimulated with 5% FCS, and oppositely, TRPM7 overexpression inhibited the phosphorylation of MEK and ERK (Figure 6C), illustrating that TRPM7 acts to suppress the activation of MEK/ERK pathway in PASMCs under this condition. To clarify the contribution of activated MEK/ERK pathway to the proliferation and apoptosis resistance increased by TRPM7 inhibition, we utilized the specific MEK inhibitor U0126 to block this pathway [32]. The results showed that the increased proliferation (Figure 6D) and apoptosis resistance (Figure 6E–6F) in PASMCs by waixenicin A treatment were completely abrogated in the presence of U0126, which efficiently blocked MEK/ERK pathway (Figure 6F). Furthermore, the proliferation (Figure 6G) and apoptosis resistance (Figure 6H–6I) increased by siRNA-mediated TRPM7 knockdown also vanished when MEK/ERK pathway was inhibited by U0126 (Figure 6I). Altogether, it could be concluded that TRPM7 inhibition- or knockdown-increased proliferation and apoptosis resistance in PASMCs rely on the activation of MEK/ERK pathway.

### TRPM7 inhibition with waixenicin A exacerbates hypoxia-induced PAH in vivo

**Figure 7:**
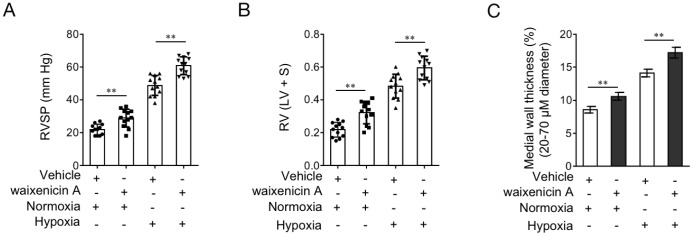
**TRPM7 inhibitor waixenicin A exacerbates hypoxia-induced PAH in vivo.** (**A**–**D**) Rats were exposed to hypoxia (10% O_2_) for 21 d or maintained under a normoxic condition, and concomitantly administrated with 200 mg/kg waixenicin A every 3 days. Each group includes 12 rats. The hemodynamic parameters including RV systolic pressure (RVSP) (**A**), and RV hypertrophy (RV/(LV+ septum (S)) (**B**) were measured. (**C**) Percentage of the medial wall thickness of pulmonary arteries. 20-70 μm in diameter. Data are mean ± SEM. n = 12. Unpaired Student’s *t*-test. **, P < 0.01 compared to vehicle control.

As last, to test whether TRPM7 inhibition reproduces PAH features in vivo, we administrated rats with waixenicin A. For examining whether waixenicin A affects disease progression, one half of rats were established with hypoxia-induced PAH. To reflect PAH features in rat model, indicators of right ventricular systolic pressure (RVSP), RV hypertrophy RV/(LV+S) and medial wall thickness of pulmonary arteries were monitored. As shown in Figure 7A–7B, waixenicin A administration significantly elevated RVSP (Figure 7A) and RV/(LV+S) (Figure 7B) in rats with or without hypoxia-induced PAH. Moreover, the medial wall thickness of pulmonary arteries in rats was also increased by waixenicin A, independent of hypoxia-induced PAH (Figure 7C). Taken together, TRPM7 inhibition with waixenicin A not only results in PAH features, but also exacerbates hypoxia-induced PAH in rats, which may highlight the important role of TRPM7 in antagonizing the development and progression of PAH.

## DISCUSSION

It’s well established that two typical traits, including the increased proliferation and apoptosis resistance, of vascular cells contribute to the progressive remodeling of pulmonary arteries and the formation of PAH. These pathological changes are reminiscent of those found in cancer cells [33], which offers an opportunity to treat PAH by exploiting therapeutic strategies used for curing cancer patients, such as imatinib and dasatinib [34–36]. The beneficial effects of these anti-neoplastic drugs on reversing the vascular remodeling process ascertain the increased proliferation and apoptosis resistance as two promising targets for clinical treatment of PAH. Motivated by these clues and the previously reported involvement of TRPM7 in promoting vascular proliferation, we aimed to investigate whether TRPM7 has a clinical relevance to PAH and explore its possible functional role and underlying mechanisms in influencing PAH features in vitro and in vivo conditions by utilizing the cultured PASMCs and a rat model with hypoxia-induced PAH. In the present study, we provide several lines of evidence showing that the downregulation of TRPM7 expression is connected to the pathogenesis of PAH and that their connection may at least partly attributed to the negative regulation of MEK/ERK pathway-mediated increase in proliferation and apoptosis resistance in PASMCs.

The conclusion of our study is based on the following evidence: 1) TRPM7 downregulation was observed in PASMCs from human with PAH and a rat model with hypoxia-induced PAH; 2) TRPM7 downregulation was reproduced in PASMCs in response to PAH stimuli in vitro; 3) TRPM7-mediated transportation of magnesium was impaired in PASMCs in response to PAH stimuli in vitro; 4) TRPM7 inhibition or knockdown reproduced and its overexpression attenuated PAH features in PASMCs in vitro, including increased proliferation and apoptosis resistance; 5) Block of MEK/ERK pathway activation abrogated the increased proliferation and apoptosis resistance in PASMCs by TRPM7 inhibition or knockdown; 6) Pharmaceutical inhibition of TRPM7 with waixenicin A induced PAH features and exacerbated the progression of hypoxia-induced PAH in rats.

We show that TRPM7 expression is decreased in PAH PASMCs at both the mRNA level and protein level. This suggests that the transcription of TRPM7 may be inhibited under this pathological condition. We guess PAH stimuli, such as growth factors and cytokines present in PAH patients, may function as extracellular stimulations that connect to intracellular machinery which restricts the transcription of TRPM7. This is very possible, since the in vitro evidence reveals that various pro-hypertensive stimuli, such as 5% FCS, IGF-1, TNF-α and IL-6, are able to decrease TRPM7 expression. Although conflicting findings were reported by a previous study using hypertension-inducing agents angiotensin II and aldosterone [37], in VSMCs of spontaneous hypertensive rats, the expression of TRPM7 was also found decreased [19]. Currently, how TRPM7 expression is regulated at a molecular level is far from being understood. It would be interesting to investigate the mechanisms underlying the regulation of TRPM7 transcription in PASMCs when subjected to pro-hypertensive stimulation. Meanwhile, the reduced TRPM7 currents and concentration of free intracellular magnesium found in PASMCs treated with PAH stimuli suggests that along with the downregulation of TRPM7, its normal function of transporting magnesium is accordingly dampened. Of note, TRPM7 is a bifunctional protein containing a C-terminus protein kinase fused to an ion channel [38], and magnesium concentration is supposed to regulate the activity of the kinase domain [39], and the kinase activity in turn affects the channel function [40]. Considering the downregulation of TRPM7 and the functional interaction between the channel and kinase domains, we conceive that in PAH PASMCs, the kinase activity of TRPM7 is very likely affected. TRPM7 kinase has been shown to phosphorylate serines and threonines located within alpha-helices of substrates, such as eEF2-k [41] and PLCγ2 [42]. Except for reducing magnesium transport function, we believe that PAH stimuli also influence cellular functions associated with TRPM7 kinase activity. Further studies are needed to address whether this is the case, which may help us to deeply understand the effect of PAH stimuli on TRPM7 activity.

Through gain-of-function and loss-of-function methods, we demonstrate that TRPM7 functions as a negative regulator of proliferation and apoptosis resistance in PASMCs. TRPM7-regulated magnesium influx affects endothelial function, inflammatory response and vascular integrity that underlie hypertension [14]. Since waixenicin A treatment and TRPM7 knockdown exert similar effects on promoting proliferation and apoptosis resistance in PASMCs, and waixenicin A acts through magnesium-dependent block of TRPM7 channels [30], and moreover, Mg2+ anaplerosis largely diminished its effects, we conclude that the increased TRPM7 proliferation and apoptosis resistance in PASMCs could be attributed to impaired magnesium transport and consequent decreased concentration of intracellular magnesium. Previous studies have associated TRPM7 effect on cell proliferation with the status of MEK/ERK pathway [20, 21]. In agreement with this, we also discovered that TRPM7 inhibition induced the activation of MEK/ERK pathway, and that the block of this pathway via U0126 abrogated TRPM7 effects on proliferation and apoptosis resistance in PASMCs, hence unveiling MEK/ERK pathway as a predominant mechanism accounting for TRPM7 function. TRPM7 inhibition via waixenicin A treatment not only induces PAH features and but also exacerbates hypoxia-induced PAH features. These results very possibly suggest an indispensable role of TRPM7 function in suppressing the development and progression of PAH, at least in a rat model, and further support a clinical relevance existing between TRPM7 downregulation and PAH development. It is tempting to test whether our findings could be reproducible in other preclinical PAH models, such as those induced by monocrotaline, mitomycin or genetically modification in rats or other species [22].

It should be noted that this study has three major limitations. First, the recruited clinical sample size is relatively small. The downregulation of TRPM7 in human PAH warrants further verification. Second, although we examined the effect of TRPM7 on PAH progression in rats through tactic of pharmacological inhibition, genetically modified animal models, including knock-out and knock-in of TRPM7, are preferably needed to validate our observations. Third, currently, we have no solid evidence to show whether other TRP channels or Mg^2+^ transporters (eg. TRPM6) are also involved in PAH pathogenesis. Further investigations are required to address this issue, which would advance our understanding of the contribution of dysregulated Mg^2+^ homeostasis to PAH.

In conclusion, these findings identify TRPM7 as a novel regulator involved in PAH pathogenesis and also relate the downregulated expression or impaired function of TRPM7 to the process of clinical PAH development. In light of the positive association between the decreased expression and impaired function of TRPM7 and acquired PAH features in vitro and in vivo, we propose that agonists of TRPM7 function or restored expression of TRPM7 might be of clinical benefit in reversing vascular remodeling and treating PAH.

## MATERIALS AND METHODS

### Human and rat PAH samples

Human explanted lung tissues of PAH patients (n = 8) and control donors (n = 6) were obtained during lung transplantation (Supplementary Table 1). The study was approved by the Ethics Committee of the First Affiliated Hospital of Zhengzhou University, and performed in accordance with the approved guidelines of the World Medical Association Declaration of Helsinki (updated version 2013). The informed consent was obtained from all subjects prior to sampling. The etiology of PAH is idiopathic, and the pathology of PAH was in accordance with Heath and Yacoub [43]. For the isolation and culture of PASMCs, human PASMCs were obtained from pulmonary arteries (<2 mm in diameter) of PAH patients or control donors. The isolation procedures were conducted as previously described in detail [44]. Briefly, the endothelium of pulmonary artery was removed with a scalpel blade, and then transferred to T75 flasks containing the Promocell Smooth Muscle Cell Growth Medium 2 (Promocell), which were further incubated in DMEM supplemented with 20% FBS when cells had adhered and monolayers were formed. For the culture of PASMCs, PASMCs were cultured in DMEM supplemented with 10% FBS and 1% penicillin/streptomycin and maintained at 37 °C in 5% CO_2_. PASMCs were used for experiments when cultured between 4 and 8 passages. Similarly, rat PASMCs were obtained from the peripheral small pulmonary arteries of 8-12-week-old male Sprague-Dawley rats as mentioned above.

### Rat PAH model

Male Sprague-Dawley rats (8-12-week-old, 200-250 g body weight) obtained from the Laboratory Animal Center of the Zhengzhou University (Zhengzhou, Henan, China) were used for establishing experimental PAH model via chronic hypoxia exposure. All rats were housed under a standard condition with 12 h/12 h light-dark cycle, 25°C temperature and 65% humidity, and allowed free access to food and water throughout experiments. The induction of PAH was conducted as described previously [45, 46]. In brief, rats were fed for 1 week under normoxia and then stochastically divided into 4 groups: (1) normoxia plus equal volume of vehicle (2.5% methanol in phosphate-buffered saline (PBS)); (2) normoxia plus 200 mg/kg waixenicin A; (3) hypoxia plus vehicle; (4) hypoxia plus 200 mg/kg waixenicin A. Twelve rats were included in each group (n = 12). Each rat received vehicle or waixenicin every 3 d via intraperitoneal injection in the morning prior to chronic hypoxia exposure, which was performed intermittently in a hypobaric hypoxia chamber (10% O_2_ concentration) for 10 h per day for consecutive 21 d. Rats in group 1 and group 2 were housed with room air. For animal welfare, all animal experiments were conducted in accordance with the guidelines approved by the Animal Care and Use Committee of the Zhengzhou University and complied with the Declaration of the National Institutes of Health Guide for Care and Use of Laboratory Animals.

### Stimulation and treatment of PASMCs

Rat PASMCs were treated with various relevant PAH stimuli in vitro as described previously [44]. Rat PASMCs were serum starved for 24 h and incubated with medium alone (control) or with medium containing 5% FCS or the following growth factors: 100 ng/ml IGF-1 (Peprotech), 20 ng/ml TNF-α (Peprotech) or 50 ng/ml IL-6 (Sigma-Aldrich) for 4, 12 or 24 h. For the inhibition of TRPM7, rat PASMCs were treated with 10 μM or 20 μM waixenicin A (Antimex Chemical Limied, 95230-65-2) for 24 h. The intracellular Mg^2+^ was restored by culturing PASMCs in medium supplemented with exogenous MgSO_4_ (Sigma). The dosage of the above reagents was determined by preliminary experiments, in which considerable effect was observed.

### qRT-PCR analysis

qRT-PCR analysis was conducted to determine mRNA levels [47]. Briefly, the total RNA were extracted from lung tissue and cell samples through the TRIzol reagent (ThermoFisher Scientific) according to the manufacturer’s instructions. Then, cDNA was transcribed with the ImProm-II Reverse Transcription System (Bio-Rad). qRT-PCR was performed using the SYBR Green Real-Time PCR Master Mixes (ThermoFisher Scientific). The specific primers for amplifying human TRPM7 (Accession: NM_017672) and rat TRPM7 (Accession: NM_053705) are listed as follows: human TRPM7 sense 5’-GGTCAGTTGGCCGTTGAATT-3’; human TRPM7 antisense 5’-TTCAGCCTTCCCATCCACAT-3’. Rat TRPM7 sense 5’-CTTTGGCCAGAGTGAAGCAG-3’; Rat TRPM7 antisense 5’-ATCAACTCTGTCCCATGCCA-3’. Expression was analyzed with the ∆Ct method. Results were normalized to those of β-actin and expressed as relative to control.

### Protein extraction and Western blotting analysis

Proteins were extracted from lung tissue and cell samples through homogenization in RIPA lysis and extraction buffer (ThermoFisher Scientific) according to the manufacturers’ instructions. After denature in SDS loading buffer, protein samples were separated by 8% or 10% SDS-PAGE and then transferred to PVDF membranes (Millipore). After block with 5% BSA diluted in TBST, the membranes were probed overnight at 4 °C with the following primary antibodies: anti-TRPM7 (Abcam, ab729), anti-β-actin (Santa Cruz, sc-81178), anti-cleaved caspase-3 (Cell Signaling Technology, 9661), anti-p-MEK 1/2 (Santa Cruz, sc-81503), anti-MEK 1/2 (Santa Cruz, sc-81504), anti-p-ERK 1/2 (Santa Cruz, sc-81492), anti-ERK 1/2 (Santa Cruz, sc-292838). After wash with TBST, the membranes were further incubated with secondary antibodies conjugated with horseradish peroxidase (HRP). Protein bands were detected by chemiluminescence with the ECL detection reagent (Amersham Biosciences). The densitometric analysis of protein bands was performed by ImageJ software [48]. Results were normalized to those of β-actin.

### Recording of TRPM7 currents

The whole-cell patch-clamp technique was used to record the currentss of TRPM7 as described previously [20]. In brief, rat PASMCs were seeded on coverslips, and after the stimulation of 5% FCS or TNF-α, coverslips were loaded onto the recording chamber and immersed in Ringer’s solution (mM) (NaCl, 145; KCl, 5.4; MgCl_2_, 1; CaCl_2_, 2; Hepes, 10; glucose, 10; pH 7.4). Whole-cell TRPM7 currentss were recorded through the Axon MultiClamp 700B Patch Clamp Amplifier (Axon Instruments, USA). The holding potential was set at 60 mV, and voltage ramps ranging from -100 mV to 100 mV and 500 ms duration were delivered at 5-s intervals after whole-cell configuration was established when the membrane was ruptured by gentle negative pressure. Data acquisition and analysis were performed using the PCLAMP 10.1 software (Molecular Devices, USA). The currents density of TRPM7 is expressed as currents amplitude/cell capacitance (pA/pF).

### Measurement of Mg^2+^ in PASMCs

The selective fluorescent probe of mag fura-2 AM was applied to measure the Mg2+ in rat PASMCs as conducted previously [49]. Briefly, cells were harvested after treatment, washed twice with Hank’s buffered saline solution, and then loaded with mag fura-2 AM (3 μM) diluted in the same buffer and incubated for 20 min at 37 °C in the darkness. Next, cells were washed twice and maintained for further 20 min in Hank’s buffered saline solution to ensure complete deesterification. The concentration of free Mg2+ was analyzed using the FluoroMax-4 Spectrofluorometer (Horiba Scientific) [50]. The excitation wavelength at 330 nM and emission wavelength at 511 nM of Fura dye were monitored. The concentration of free Mg2+ was calibrated to a standardized curve made according to Rmin and Rmax values obtained by measuring the sequential addition of 25 mM MgSO4 diluted in buffer containing 0.05% Triton and 50 mM EDTA.

### Assessment of cell proliferation and apoptosis

The effect of treatment on cell proliferation of rat PASMCs was assessed by BrdU incorporation assay using the BrdU Staining Kit (Laizee Biotech, Shanghai, China) following the manufacturers’ instructions. The apoptosis of rat PASMCs was assessed by TUNEL assay using the TUNEL Apoptosis Detection Kit (FITC) (YEASEN, Shanghai, China).

### Adenovirus-mediated overexpression and siRNA transfection

For overexpressing TRPM7 in rat PASMCs, adenovirus encoding the rat TRPM7 (Accession: NM_053705) (Ad-TRPM7) were packaged. Adenovirus encoding the control cDNA GFP (Ad-GFP) were used as controls. PASMCs were seeded at ~70% confluency, and then infected with adenovirus for 24 h. Cells were cultured for further 48 h in serum-containing medium to achieve the overexpression. To knockdown TRPM7 expression in rat PASMCs, cells were transfected with two different sequence of siRNAs targeting rat TRPM7 (siTRPM7#1 and siTRPM7#2) using the Lipofectamine RNAiMAX Reagent (ThermoFisher Scientific, 13778100) according to the manufacturers’ instructions. siRNA with a scramble sequence was used as negative control (siNC). The final concentration of siRNA was 25 nM. Cells were cultured for further 48 h to achieve the effect of knockdown. The sequence of siRNAs was listed as follows: siTRPM7#1 (sense, 5’-GCUGUUAAAUAGUGUUUAAAG-3’; antisense,5’-UUAAACACUAUUUAACAGCUU-3’); siTRPM7#2 (sense, 5’-GCAGAGAUAAGCUGUUAAAUA-3’; antisense, 5’-UUUAACAGCUUAUCUCUGCGG-3’); siNC (sense, 5’-UUCUCCGAACGUGUCACGUTT-3’; antisense, 5’-ACGUGACACGUUCGGAGA ATT-3’).

### Hemodynamics, RV hypertrophy and histology

Hemodynamic parameters including RVSP and SAP in rats were measured as previously described [51]. In brief, rats were anesthetized with isoflurane and then placed on a homeothermic plate (TianCheng Medical Co. Ltd., Beijing, China) in a supine position. Next, rats were tracheotomized and then ventilated using the SAR-830/AP small animal ventilator (CWE Incorporated, USA) set with a a frequency of 60 breaths/min and positive end-expiratory pressure (PEEP) (1 cm H_2_O). SAP parameter was measured by inserting the Millar PVR-1045 catheter (Millar Instruments, USA) into the left carotid artery (LCA). Additionally, RVSP parameter was measured by inserting the single lumen PE50 tubing fluid-filled catheter (ADInstruments, Australia), connected to a fluid-filled pressure transducer, into the RV via the right jugular vein. For measuring RV hypertrophy and histology, rats were then exsanguinated, and the hearts and lungs were harvested. The left lung was fixed for histology with 4% paraformaldehyde in PBS before dehydration and paraffin embedding. Right ventricular hypertrophy was determined as the ratio of right ventricular to left ventricular and septal weight (RV/LV+ S) from the tissue. The medial wall thickness of pulmonary arteries was measured by the elastin-stained left lung sections performed as previously documented [52].

### Statistical analysis

All data are expressed as the mean ± SD, except for hemodynamic parameters and medial wall thickness, which are expressed as the mean ± SEM. The D’Agostino and Pearson omnibus normality test was used for testing data normality. Statistical analysis was performed with the GraphPad Prism 6.0 Software by using the unpaired Student’s *t*-test for comparing data from two groups. One-way ANOVA test was applied to compare data from more than two groups. *P* values less than 0.05 were considered to be statistically significant.

## Supplementary Material

Supplementary Figures

Supplementary Table 1
