# Supplementary material for: TRPM7 channel inhibition exacerbates pulmonary arterial hypertension through MEK/ERK pathway
Source: Aging (Albany NY). 2019 Jun 19;11(12):4050–65. doi: 10.18632/aging.102036 (PMC6629001; doi:10.18632/aging.102036)
Supplement: Supplementary Figures [file aging-11-102036-s002.pdf]

SUPPLEMENTARY FIGURES

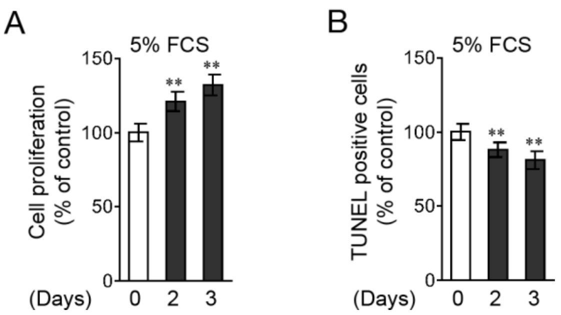

Supplementary Figure 1. Increased proliferation and apoptosis resistance in PASMCs following 5% FCS stimulation. Rat PASMCs were serum starved for 24 h, followed by incubation with medium alone (control) or containing 5% fetal calf serum (FCS) for 2 or 3 days. (A) The cell proliferation was determined by BrdU incorporation assay. (B) Cell apoptosis was detected by TUNEL staining. Results are expressed as a percentage relative to control. Data are mean ± SD. n = 3. One-way ANOVA test. **, P < 0.01 compared to control.

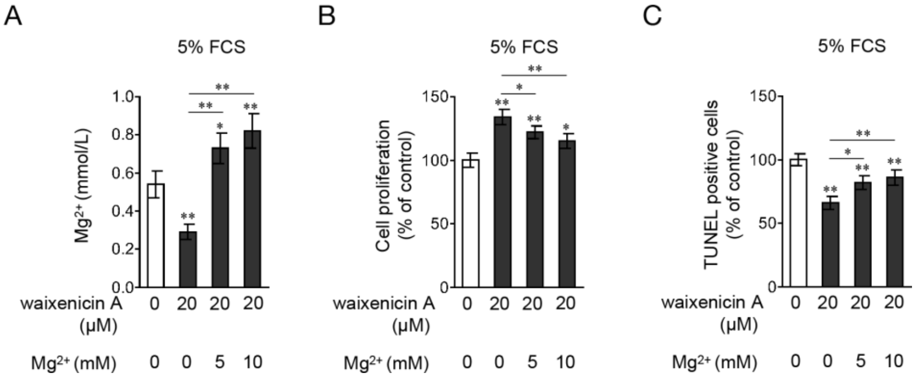

Supplementary Figure 2. Mg2^{+} anaplerosis largely rescues effects of TRPM7 inhibition on proliferation and apoptosis resistance in PASMCs. Rat PASMCs were serum starved for 24 h, followed by incubation with medium containing 5% FCS for 24 h in the presence or absence of 20 μM waixenicin A, or 5 mM or 10 mM Mg2^{+} as indicated. (**A**) Intracellular free Mg2 concentration was determined. Data are mean ± SD from 3 independent experiments. (**B**) The cell proliferation was determined by BrdU incorporation assay. (**C**) Cell apoptosis was detected by TUNEL staining. Results are expressed as a percentage relative to control. Data are mean ± SD. n = 3. One-way ANOVA test. **, P < 0.01; *, P < 0.05 compared to control.

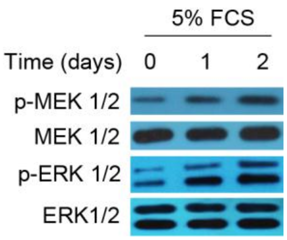

Supplementary Figure 3. The MEK/ERK pathway is activated in PASMCs stimulated with 5% FCS. Rat PASMCs were serum starved for 24 h, followed by incubation with medium alone (control) or containing 5% FCS for 1 or 2 days. The protein expression of p-MEK 1/2, MEK 1/2, p-ERK 1/2 and ERK 1/2 was determined by Western blot analysis. The representative images from 3 independent experiments are shown.
