# Supplementary material for: TRPM7 channel inhibition exacerbates pulmonary arterial hypertension through MEK/ERK pathway
Source: Aging (Albany NY). 2019 Jun 19;11(12):4050–65. doi: 10.18632/aging.102036 (PMC6629001; doi:10.18632/aging.102036)
Supplement: Supplementary Table 1 [file aging-11-102036-s001.pdf]

SUPPLEMENTARY TABLE

Supplementary Table 1. Clinical characteristics of healthy donors and PAH patients.

	Healthy controls (n = 6)	PAH patients (n = 8)	Significance
Age (years)	52 ± 11.3	55 ± 14.5	NS
Male (%)	50	50	NS
BMI (kg/m2)	25.7 ± 3.9	26.3 ± 4.2	NS
Smoking history (%)	25	33	NS
mPAP (mmHg)	–	57 ± 17	–
PVR (Wood units)	–	7.9 ± 2.6	–

Note: BMI, body mass index; mPAP, mean arterial pulmonary pressure; PVR, pulmonary vascular resistance; NS, not significant
